# The impact of the COVID-19 pandemic on Fabry Disease Patients: an examination of Mood Status, Therapy Adherence, and COVID-19 infection

**DOI:** 10.1186/s13023-022-02491-7

**Published:** 2022-09-05

**Authors:** Cebrail Karaca, Mevlut Tamer Dincer, Seyda Gul Ozcan, Betul Sarac, Saffa Ahmadzada, Selma Alagoz, Alev Bakir, Ertugrul Kiykim, Sinan Trabulus, Nurhan Seyahi

**Affiliations:** 1 Department of Nephrology, Cerrahpasa Medical Faculty, Istanbul University - Cerrahpasa, Kocamustafapaşa St. No: 53, Fatih, 34360 Istanbul, Turkey; 2 Department of Internal Medicine, Cerrahpasa Medical Faculty, Istanbul University - Cerrahpasa, Istanbul, Turkey; 3 Department of Pediatric Nutrition and Metabolism, Cerrahpasa Medical Faculty, Istanbul University - Cerrahpasa, Istanbul, Turkey; 4 Department of Nephrology, Istanbul Training and Research Hospital, University of Health Sciences, Istanbul, Turkey; 5 Department of Social Pediatrics, Institute of Child Health, Istanbul University, Istanbul, Turkey

**Keywords:** COVID-19, Enzyme replacement therapy, Fabry disease, Psychological effect

## Abstract

**Background:**

Fabry disease (FD) is a rare metabolic disorder, in which a lifelong enzyme replacement therapy (ERT) constitutes the cornerstone of disease-specific therapy. In this study, we examined the effects of the COVID-19 pandemic and lockdown measures on the management of FD patients.

**Methods:**

We collected data in three main domains; mood status, adherence to ERT, and COVID-19 infection. We used the Hospital Anxiety and Depression Scale (HADS) to evaluate the mood statuses of FD patients and the Morisky Medication Adherence Scale (MMAS) and the Medication Adherence Report Scale (MARS) to assess patients’ adherence to non-disease specific therapy. We also examined a control group to compare the mood status data.

**Results:**

A total of 67 FD patients (males: 47.8%, mean age: 37.0 years) were recruited to the study, of which 58 were receiving ERT. Both the HADS depression and anxiety scores were higher in the control group compared to FD patients. During the first wave of the pandemic, 25 patients reported to have missed an infusion for a mean of 2.3 ± 1.7 doses and half of the patients had adopted a home-based infusion treatment regimen. COVID-19 infection developed in 25 patients, of which one died. The majority of our patients (71.6%) have had at least one shot of the vaccine.

**Conclusion:**

We found that FD patients were more resilient to the negative psychological effects of lockdown. Traumatic growth may be an important factor in explaining this finding. Government-supported home therapy programs might be beneficial for FD patients to increase the therapy adherence.

## Introduction

Fabry disease (FD) is a progressive, multisystemic, X-linked inherited lysosomal storage disease caused by pathogenic mutations in the α-Galactosidase A (GLA) gene. Complete or partial deficiency of the GLA enzyme activity leads to progressive deposition of glycosphingolipids, particularly globotriaosylceramide (Gb3/GL3) and its deacylated derivative globotriaosylsphingosine (lyso-Gb3/lyso-GL3) in various organs [1]. In Turkey, enzyme replacement therapy (ERT) with recombinant agalsidase alpha or beta are administered in health facilities.

On March 11, 2020, the World Health Organization (WHO) announced the pandemic status for a new type of coronavirus (severe acute respiratory syndrome coronavirus 2 [SARS-CoV-2]) infection (COVID-19) [2]. Different measures have been taken in various countries to ensure that the patient care capacities of health facilities are not exceeded. Among these, in our country, curfews were imposed on individuals over the age of 65 and under the age of 18, and schools, cafeterias, movie theaters, gyms, and restaurants were closed. The curfew was maintained on weekends and public holidays, except for mandatory situations. Outpatient clinics were reduced except for emergency cases [3].

Fabry disease patients with severe organ involvement such as kidney, or heart failure should be considered as high-risk patients regardless of their age [4, 5]. Therefore, adherence to curfew measures should be advised to FD patients, while avoidance of hospital access might cause them to skip ERT doses. Additionally, the pandemic and pandemic control measures might increase stress, anxiety, and depression. Those mood states might decrease the willingness of FD patients to receive ERT [5].

In this study, we aimed to evaluate the impact of the COVID-19 pandemic on FD patients with special emphasis on mood status changes, adherence to ERT, and COVID-19 infection.

## Methods

### Study design and participants

We collected data from FD patients who were under regular follow-up in the nephrology and pediatric nutrition and metabolism departments of our tertiary care university hospital between June 2020 and November 2021. Patients who declared their will to participate in the study during a preliminary phone interview were included in the study.

The data were collected on three domains. First, we inquired the skipped doses of ERT for the period between April and June 2020. During this period, the most severe restrictions related to pandemic were in effect. In addition, we assessed the patients’ compliance with non-Fabry-related drugs using the Medication Adherence Report Scale (MARS) and the Morisky Medication Adherence Scale (MMAS). Second, between June and September 2020, we collected information about patients’ behavior during the pandemic with a standard questionnaire that we used in our previous studies and assessed their mood status using the Hospital Anxiety and Depression Scale (HADS) [6–8]. Finally, between August and November 2021, we collected data on COVID-19 infection and vaccination status of the patients. The questionnaires were filled in via telephone interviews. Additionally, we recorded the clinical and laboratory data from the medical records of the patients. The diagnosis of COVID-19 was confirmed with at least one positive real-time reverse transcriptase-polymerase chain reaction (RT-PCR) test result.

To obtain comparative data about the mood status during the lockdown period, we also collected data from healthy subjects using the same electronic survey forms filled by the patients. The control group consisted of individuals without a self-reported chronic disease. We used a snowball sampling strategy to recruit subjects to the control group. We selected age-, gender-, and educational status-matched subjects to construct the control group.

### Questionnaires

#### Hospital Anxiety and Depression Scale

The HADS is commonly used to assess the anxiety and depression of patients [9]. The term “hospital” in its title suggests that it is used for patients only, but many studies have confirmed that it can be used in community settings and primary care medical practice, too [10]. The HADS contains two subscales that measure symptoms of depression (HADS-D; 7 items) and anxiety (HADS-A; 7 items) during the previous week. The items are scored on a four-point scale from 0 to 3; and for each subscale, the total score is at most 21. In our study, we used the validated Turkish version of the HADS. The HADS scores can be interpreted using cut-off values. For the Turkish version, a cut-off value of 10 for the HADS-A score and a cut-off value of 7 for the HADS-D score was proposed to classify the scores as ‘abnormal’ [11].

#### Medication Adherence Report Scale

The MARS provides a short and accurate assessment of drug compliance. It consists of five questions expressing negative drug compliance behavior (deciding to skip the drug dose, forgetting to take the drug, changing the drug dose, stopping the drug for a while, and under-dosing take) [12]. The scale has been used to evaluate drug compliance in many chronic diseases. Each question is scored as 5: never, 4: rarely, 3: sometimes, 2: often, 1: very often, and the total score ranges between 5 and 25. An increase in the total score indicates drug compliance, while a decrease indicates otherwise. In our study, we used the validated Turkish version of the MARS [13].

#### Morisky Medication Adherence Scale

The MMAS is a self-reported scale measuring adherence to medication and consists of four close-ended questions with two choices as “yes/no”. If all questions are answered as “no”, drug compliance is considered high, if one or two questions are answered as “yes”, drug compliance is considered as moderate, and if three or four questions are answered as “yes”, drug compliance is considered as low [14]. In our study, we used the validated Turkish version of the MMAS [15].

### Statistical analysis

Descriptive statistics were expressed as mean, standard deviation (SD), median, minimum, and maximum for the continuous data, and as count and proportion for the categorical data. The categorical data were analyzed using the chi-square or Fisher’s exact tests. The normality of the continuous variables was calculated using the Shapiro–Wilk test. The normally distributed continuous variables were compared using the independent samples *t*-test, while the Mann–Whitney U test was employed for the non-normally distributed data. Statistical analyses were performed using the IBM SPSS for Windows v.24 software and were reported with 95% confidence intervals. Values of *p* < 0.05 were considered significant.

## Results

### Demographic, clinical, and laboratory data

After eliminating three patients who could not fill in the questionnaires, 67 out of the contacted 70 patients were included in the study. Demographic, clinical, and laboratory data of the participants are shown in Table 1. Fabry disease patients were generally young to middle-aged (38.8 ± 13.2 years) and nearly half of them were males (47.8%). The prevalences of hypertension, chronic obstructive pulmonary disease, and diabetes mellitus were 30.3%, 7.5%, and 4.5%, respectively. Eight patients (11.9%) were on renal replacement therapy, six were undergoing hemodialysis, and two have received a kidney transplant. The age and gender distribution of the controls were similar to those of the FD patients (mean age: 40.6 ± 14.4 years, males: 47.8%).

**Table 1:** Demographic, clinical, and laboratory data of Fabry disease patients

Characteristics	Patients (*n* = 67)
Age, years	37.0 ± 11.8
Male gender, %	47.8
Clinical manifestation of Fabry disease, %	
Skin findings	17.9
Hearing loss	20.9
Cornea verticillata	50.7
Heart involvement	43.2
Stroke	16.4
Acroparesthesia	67.1
Kidney involvement	65.7
Lyso-Gb3, ng/ml	10.7 ± 11.5
Median (min–max)	6.3 (0.5–42.2)
Enzyme replacement therapy, %	86.6
Agalsidase alfa	75.9
Agalsidase beta	24.1
ERT duration, years	4.6 ± 3.7

Data are expressed as mean ± SD for quantitative parameters and n (%) for nominal parameters unless otherwise stated

ERT, enzyme replacement therapy

### Sociocultural data and mood status

**Fig. 1:**
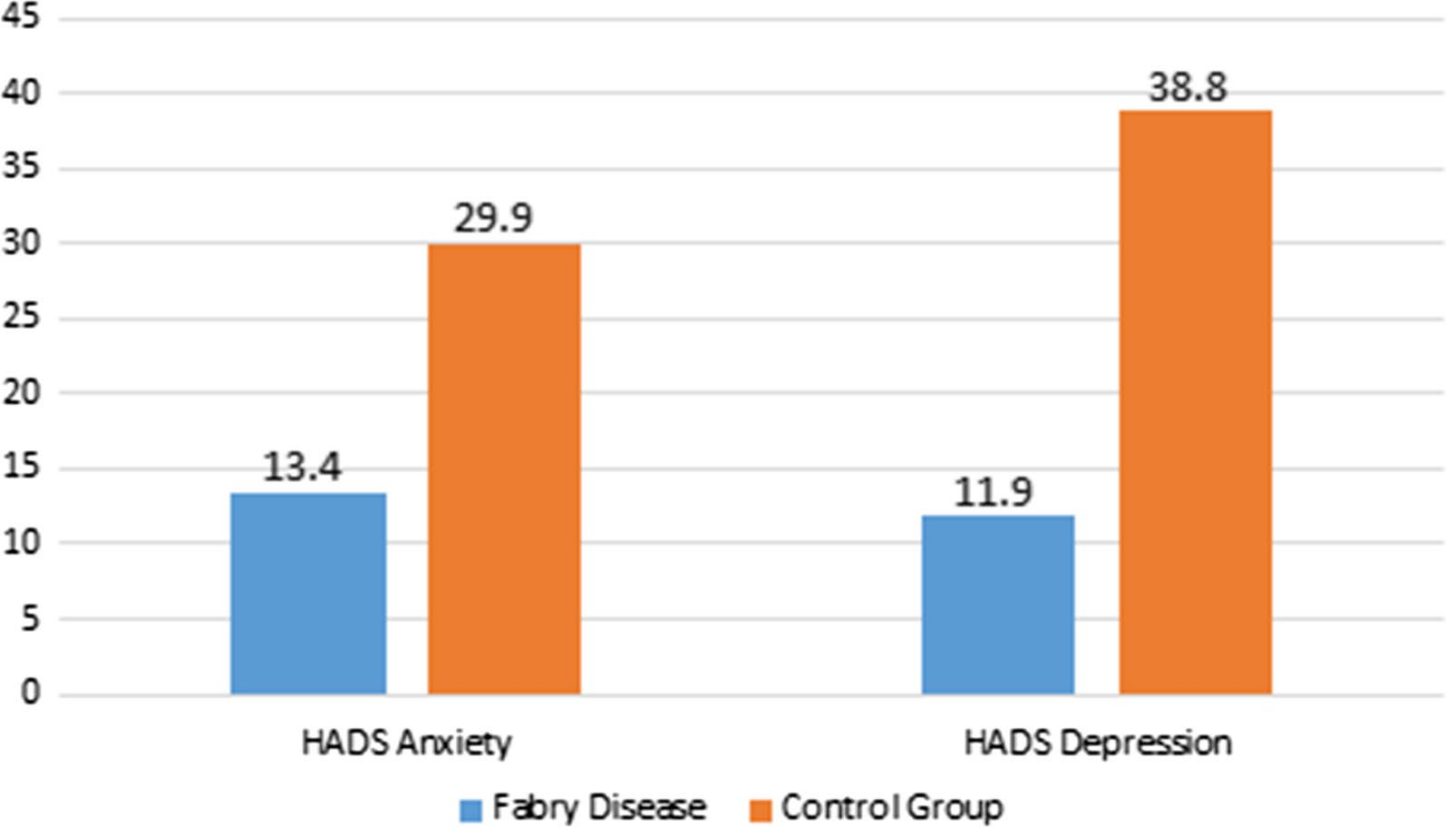
The percentage of the patients classified as abnormal according to the HADS anxiety and HADS depression scores for each study group (HADS anxiety: *p* = 0.036, HADS depression: *p* = 0.001). HADS, Hospital Anxiety and Depression Scale

Sociocultural data of FD patients and the control group are shown in Table 2. Most of the FD patients were married and had children. Regarding sociocultural characteristics, there were no significant differences between FD patients and control group except for the consideration of the seriousness of the outbreak which was higher in FD patients and the feeling of being obliged to leave home for work, which was higher in the control group. The HADS scores for both groups based on the anxiety and depression subscales are shown in Table 3. Both the HADS anxiety and HADS depression scores were significantly lower in FD patients compared to the control group. The stratification of the study groups according to gender is also shown in Table 3. In both male and female FD patients, both the HADS anxiety and the HADS depression scores were significantly lower compared to the control group. The percentage of the patients classified as abnormal according to the HADS anxiety and HADS depression scores were significantly lower in the FD group (Fig. 1).

**Table 2:** Sociocultural data of Fabry disease patients and controls

Characteristics	Patients (n = 67)	Controls (n = 67)	*p*
Marital status (married), %	71.6	68.7	0.706
Have children (yes), %	62.7	68.7	0.467
Level of education, %			
Primary school	55.2	52.2	
Highschool	17.9	20.9	0.901
University or higher	26.9	26.9	
Living with spouse and children, %	77.6	73.1	0.547
Household size, %			
1–3	46.3	58.2	
4–6	49.2	37.3	0.365
> 6	4.5	4.5	
Consider the outbreak, %			
Very serious	76.1	55.2	0.011
Serious	23.9	44.8	
Follow the advice to stay home (yes), %	97	91	0.274
Feel obliged to leave home for work? (yes), %	11.9	40.3	< 0.001
Think that they have received adequate medical support during the pandemic period (yes), %			
	58.2	53.7	0.602

Data are expressed as mean ± SD for quantitative parameters and n (%) for nominal parameters unless otherwise stated

**Table 3:** The Hospital Anxiety and Depression Scale scores of the study groups

	Fabry disease	Control group	*p*
All study subjects	(n = 67)	(n = 67)	
HADS anxiety score	4.9 ± 3.6 (4.0)	8.0 ± 4.5 (8.0)	< 0.001
HADS depression score	4.3 ± 3.3 (4.0)	6.9 ± 4.0 (7.0)	< 0.001
Males	(n = 32)	(n = 38)	
HADS anxiety score	4.1 ± 4.0 (3.0)	7.8 ± 5.2 (8.0)	0.001
HADS depression score	4.2 ± 4.0 (3.0)	7.0 ± 4.5 (6.0)	0.004
Females	(n = 35)	(n = 29)	
HADS anxiety score	5.8 ± 3.1 (5.0)	8.3 ± 3.6 (8.0)	0.004
HADS depression score	4.5 ± 2.8 (4.0)	6.8 ± 3.6 (7.0)	0.006

Data are expressed as mean ± SD and (median)

HADS, Hospital Anxiety and Depression Scale

### Therapy adherence

Enzyme replacement therapy-related characteristics of FD patients are shown in Table 4. Before the pandemic, the majority of the patients were used to receiving their ERT in hospitals. During the pandemic, the most striking difference was that more than half of the patients have received ERT at home. Twenty-four FD patients (41.4%) were found to have skipped their ERT sessions during the pandemic period, at least one dose, between April and June 2020. The main reason behind this was failing to go to hospital due to the fear of getting infected (62.5%). Nine patients did not develop any complaints, whereas nine complained of neuropathic pain, three complained of tinnitus, two patients complained of abdominal pain/diarrhea, and one patient complained of headache.

**Table 4:** Enzyme replacement therapy-related characteristics of Fabry disease patients

Characteristics	Patients (n = 58)
Before the pandemic	
Where did you receive ERT?, %	
Health clinic	5.2
Private hospital	8.6
Public hospital	56.9
University hospital	28.3
During the pandemic	
Have you received ERT regularly during the pandemic? (no), %	41.4
If you haven't, why?, %	
Afraid to go to the hospital due to fear of contagion	62.5
Inability to find a hospital for the infusion	25
Lack or inability to obtain medication	12.5
How many doses have you skipped?	2.3 ± 1.7
Median (min–max)	1.5 (1–6)
Where did you receive ERT?	
Health clinic	1.7
Private hospital	13.8
Public hospital	31
University hospital	1.7
Home	51.7

Data are expressed as mean ± SD for quantitative parameters and n (%) for nominal parameters unless otherwise stated

ERT, enzyme replacement therapy

**Fig. 2:**
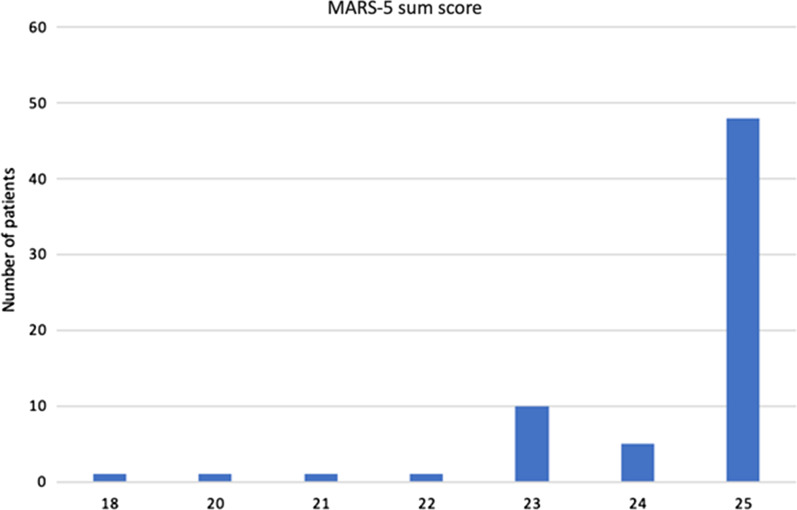
Distribution of the MARS scores among Fabry disease patients for non-ERT drugs. The mean MARS score was 24.3 ± 1.3, while the median was 25 (range: 18–25). MARS, Medication Adherence Report Scale

Fabry disease patients were using additional, non-ERT drugs for the following conditions; cardiovascular diseases (31.3%), hypertension (29.9%), chronic kidney disease (22.4%), stroke (10.4%), and other diseases (25.5%). A summary of the MARS scores in FD patients for those drugs are shown in Fig. 2. The mean score was 24.3 ± 1.3, which can be inferred as that the therapy adherence for non-ERT drugs was acceptable. The MMAS score was 1 in 83.6% and 2 in 16.4% of the patients, which implicated a moderate adherence to therapy.

### COVID-19 infection and vaccination

All patients were questioned for history of COVID-19 infection between March 2020 and November 2021, and 37.3% were found to have been infected with COVID-19. These patients were usually young to middle-aged (36.6 ± 12.4 years) and female (60%) in majority. The patients showed symptoms of myalgia (80%), cough (72%), smell and taste loss (68%), fever (52%), headache (40%), dyspnea (24%), nausea vomiting and diarrhea (16%) at admission. All patients had a family member infected with COVID-19 except for one. Typical COVID-19 pneumonia was observed in the thorax CT of all patients except two. Five patients had to be hospitalized for the management of FD (Table 5) [16]. One of them had to be followed up in the intensive care unit and later died. These 5 patients hospitalized for COVID-19 infection did not receive any COVID-vaccine dose. Favipiravir was used in 76%, hydroxychloroquine in 4%, and corticosteroids in 8% of the patients. Anticoagulant drugs were used in 16% of the patients.

**Table 5:** Characteristics of FD patients hospitalized for COVID-19 infection

	#1	#2	#3	#4	#5
Age	45	36	42	65	54
Gender	M	M	M	F	M
Fabry phenotype	Classic	Classic	Classic	Classic	Classic
Fabry-related risk factor	LVH, Kidney tx	LVH, stroke	LVH, stroke	LVH, stroke	LVH, stroke
ERT	Yes	Yes	Yes	Yes	Yes
COVID-19 related variables					
Symptoms	Fever, cough, dyspnea, myalgias	Cough, myalgias, diarrhea	Cough, headache	Fever, cough, dyspnea	Fever, cough, dyspnea, ageusia and anosmia
Disease severity*	Severe	Moderate	Moderate	Moderate	Critical
Treatment	Favipiravir, Corticosteroid LMWH	Favipiravir, LMWH	Favipiravir, LMWH	Favipiravir, LMWH	Favipiravir, Corticosteroid LMWH
Vaccination status	No	No	No	No	No
Outcome	Recovered	Recovered	Recovered	Recovered	Died

ERT, Enzyme replacement therapy; M, Male; F, Female; Kidney tx, Kidney transplantation; LVH, Left ventricular hypertrophy; LMWH, Low-molecular weight heparin

*Disease severity was classified according to reference 16

In examination of major organ involvements in five FD patients who were hospitalized for COVID-19, four of them were found to have a heart, kidney, and CNS involvement and one heart and kidney involvement. One of the patients with kidney involvement underwent renal transplantation and one was on hemodialysis treatment. The FD patient received a kidney transplant in 2014 and was on triple immunosuppressive therapy at the time of infection consisting of steroids, tacrolimus, and mycophenolate mofetil. Out of the 25 patients with COVID-19, 21 (84%) were on ERT, but 10 (47.6%) of them have skipped their ERT sessions during the infection period. The number of missed ERT doses was 3.1 ± 2.2.

When asked about their vaccination status, 56 of the 67 patients replied, revealing that 48 of them (71.6% of the total cohort) were vaccinated. Most of the patients have received two shots of the vaccine (60.4%), while 29.3% have received three shots and 10.4% one shot. Myalgia was the most commonly reported complaint (10.7%, six patients) followed by fever (two patients). Post-vaccination infection was reported only by one patient.

## Discussion

In this study, we examined the effect of the COVID-19 pandemic on FD patients from different aspects. Our findings have implications on the management of FD patients, which should be further discussed. First, we found that FD patients were more resilient to the effects of lockdown measures compared to the healthy controls. Secondly, FD patients skipped their ERT sessions during the pandemic period because of pandemic related restrictions or because of COVID-19 infections. Finally, more than 35% of the patients had been infected with COVID-19, experiencing a relatively mild course.

Similar to the findings from previous studies performed on patients with chronic diseases, we also found that FD patients have psychological resilience to the effect of lockdown measures [6–8]. Negative psychological effects of lockdown measures and social distancing have been well documented [6–8, 17]. Posttraumatic growth along with familiarity with medical issues were suggested as major factors explaining the psychological resilience that we observed in FD patients [8]. Beside psychological growth, FD patients frequently visit the hospital and interact with doctors, nurses, and fellow patients. They become familiar with medical concepts and complications Therefore, “the fear of the unknown” might be less than for the healthy population. Finally, they have easier access to their physicians or nurses at least by tele-medicine means as they are closely followed because of their special rare disease. All those factors might be associated with the observed psychological resilience of FD patients compared to controls.

The COVID-19 pandemic resulted in interruption of ERTs in various lysosomal storage diseases (LSDs) including FD [18, 19]. We found that the fear of going to hospital was the most common cause of missing the ERT sessions. Similar results were reported by others [19, 20]. Home-based treatment was suggested as a viable option to prevent treatment disruptions [19, 21]. The effects of temporary discontinuation of ERT were studied in other LSDs such as Gaucher disease [22, 23]. In line with this observation, it has been suggested that ERT should not be discontinued in FD patients during the pandemic [24]. Twenty-four of our patients have missed 2.3 ± 1.7 doses on average because of the pandemic control measures. In a recent study conducted on LSD patients, it was pointed out that missing two or more doses of therapy led to worsening of the symptoms [19]. Treatment discontinuation was associated with fast deterioration of the organs and new Fabry manifestations [25]. As a solution to this problem, some of our patients have started receiving home treatment using their own resources. Obviously, social security-based regulations might provide a better means for home-based therapy. There are different regulations across Europe regarding home-based ERT in FD patients [21, 26]. Especially, experience from Italy shows favorable effects of this modality in FD patients [27]. In Turkey, home-based ERT infusion is not routinely available. However, during the pandemic, regular drug supply was ensured for FD patients; the validity periods of the medication reports were extended and ERT drugs were provided without a new prescription. Beside pandemic control measures, COVID-19 infection was another reason for missing the ERT sessions. For this reason, ten patients missed 3.1 ± 2.2 doses on average.

As expected, the cumulative incidence of COVID-19 cases increased with the prolonged follow-up period. A similar finding was previously reported for another patient cohort with Behcet’s disease from Turkey [28]. It is hard to obtain comparative data from the general population. However, the cumulative incidence of PCR-confirmed COVID-19 cases in Istanbul (the largest city of Turkey with a population of 15.8 million) was estimated as 11.2% (95% CI, 1.9–13.2) as of April 2021 [29, 30]. Similar to patients with Behcet’s disease, the cumulative incidence of COVID-19 in FD patients seems to be higher than that of the general population. We want to point out that in addition to ascertainment bias, longer exposure in our cohort might be responsible for this observation. Additionally, FD patients are more likely to visit high-risk places, such as hospitals, for ERT.

The majority of our patients (71.6%) have had at least one shot of the vaccine. This finding is in parallel with that of the general population as reported by the Ministry of Health [31]. Vaccination might be a reason for the relatively mild course of COVID-19 in our patients. There are previous reports of mild COVID-19 infection in Fabry disease patients [32]. Additionally, Ghosh et al. suggested that a lysosomal storage in general may prevent the occurrence of a severe COVID-19 infection [33].

Our cross-sectional study had several limitations. First, the data regarding the mood status were collected during a period of two months. Therefore, our study group may not be homogeneous regarding the effect size. Second, information bias related to errors in self-reported data should be kept in mind. Additionally, we evaluated the ERT parameter for a period of three months. However, this period was also the time with the most severe lockdown measures. Finally, countries had different lockdown measures and therapy options. Therefore, the generalizability of our results might be limited.

In conclusion, FD patients who are more resilient to negative psychological effects of lockdown should be advised to strictly adhere to infection control measures. Home-based therapies should be reinstituted to avoid ERT disruptions.

## Data Availability

Data is available upon request.
